# Akutes vestibuläres Syndrom mit cochleärer Beteiligung – ein neurologischer Notfall?

**DOI:** 10.1007/s00106-023-01403-w

**Published:** 2024-01-03

**Authors:** David Bächinger, Alexander A. Tarnutzer, Ralf Gold, Carsten Lukas, Stefan Dazert, Julia Dlugaiczyk

**Affiliations:** 1 Klinik für Hals‑, Nasen- und Ohrenheilkunde, St. Elisabeth-Hospital, Ruhr-Universität Bochum, Bochum, Deutschland; 2 Klinik für Ohren‑, Nasen‑, Hals und Gesichtschirurgie, Universitätsspital Zürich, Rämistrasse 100, 8091 Zürich, Schweiz; 3 Universität Zürich, Zürich, Schweiz; 4 Klinik für Neurologie, Kantonsspital Baden, Baden, Schweiz; 5 Neurologische Universitätsklinik, St. Josef Hospital, Ruhr-Universität Bochum, Bochum, Deutschland; 6 Institut für Neuroradiologie, St. Josef Hospital, Ruhr-Universität Bochum, Bochum, Deutschland

## Anamnese

Ein 66-jähriger Patient stellte sich notfallmäßig mit seit zwei Tagen bestehendem konstantem Drehschwindel ohne erinnerlichen Auslöser vor. Begleitend sei eine Hörminderung auf dem linken Ohr aufgetreten. Weiter bestehe seit Jahren ein bilateraler Tinnitus, welcher sich nicht verstärkt habe. Auf Nachfrage äußerte der Patient keine Ohrschmerzen, keine Otorrhö, keine Fühlstörungen oder Parästhesien, keine motorischen Störungen der Extremitäten, keine Dysphagie, keine Dysarthrie, keine Kopfschmerzen und keine Sehstörungen. An Nebendiagnosen bestanden ein Diabetes mellitus Typ 2, eine arterielle Hypertonie sowie ein Z. n. benignem paroxysmalem Lagerungsschwindel vor Jahren. Als Dauermedikation nahm der Patient Empagliflozin, Sitagliptin und Metoprolol ein. In der Noxenanamnese fand sich weder ein Alkoholkonsum noch ein Nikotinabusus.

## Klinischer Befund

Der Patient präsentierte sich in gutem Allgemeinzustand, afebril, normoton und normokard. Er wies einen horizontalen Spontannystagmus Alexander-Grad III nach rechts mit torsioneller Komponente auf. Der Nystagmus war verstärkt unter der Frenzel-Brille sowie bei Blick nach rechts (Alexander-Gesetz). Im klinischen Kopfimpulstest für die lateralen Bogengänge zeigte sich eine deutliche Rückstellsakkade bei Kopfdrehung nach links, nach rechts fiel der Kopfimpulstest unauffällig aus. Beide Ohren waren äußerlich unauffällig. Ohrmikroskopisch fand sich beidseits ein reizloser Gehörgang sowie ein reizloses, differenziertes und bewegliches Trommelfell. In den Stimmgabelversuchen lateralisierte der Weber-Test nach rechts, der Rinne-Versuch wurde links nicht gehört und war rechts positiv. Im wechselseitigen Abdecktest fand sich keine vertikale Blickdeviation („Skew Deviation“). In der okulomotorischen Untersuchung zeigten sich glatte – vom Spontannystagmus überlagerte – horizontale und vertikale Augenfolgebewegungen, eine normale Konvergenzreaktion und eumetrische horizontale und vertikale Sakkaden. Im Romberg-Test demarkierte sich eine ausgeprägte Fallneigung nach links, wobei das selbstständige Stehen und Gehen auch ohne Unterstützung noch möglich war. Es bestand kein fokalneurologisches sensibles oder motorisches Defizit, keine Dysmetrie im Finger-Nase-Versuch und keine Dysdiadochokinese.

## Audiovestibuläre Diagnostik

**Abb. 1:**
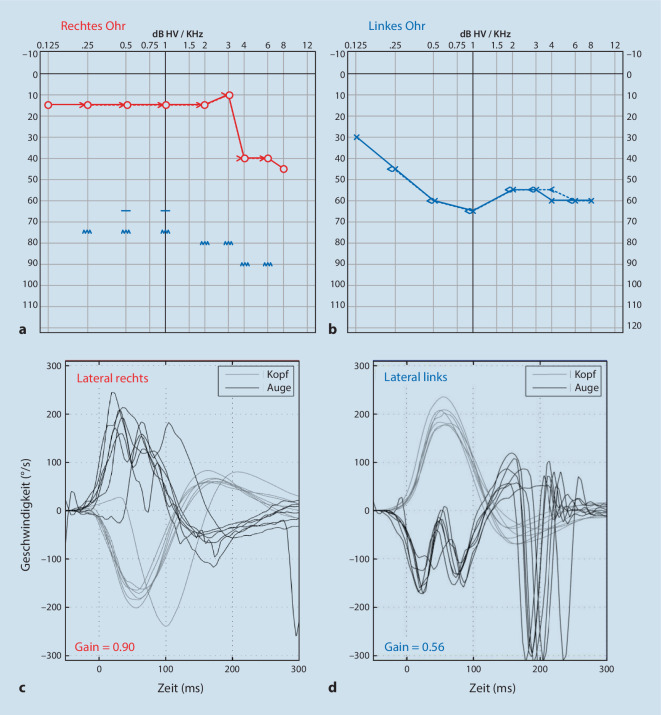
Audiovestibuläre Diagnostik. **a,** **b** Im Reintonaudiogramm zeigte sich rechts eine leicht- bis mittelgradige sensorineurale Schwerhörigkeit im Hochtonbereich, vereinbar mit einer Presbyakusis (**a**). Links fand sich eine pantonale mittel- bis hochgradige sensorineurale Schwerhörigkeit (**b**). **c,** **d** Im Video-Kopfimpulstest der lateralen Bogengänge zeigte sich rechts ein normaler Verstärkungsfaktor des vestibulookulären Reflexes (Gain = 0,90; **c**). Links fand sich ein verminderter Verstärkungsfaktor des vestibulookulären Reflexes (Gain = 0,56) mit deutlichen Overt-Rückstellsakkaden (**d**). Die M‑förmige Deformation der Kurven der langsamen Augenbewegungen beidseits ist durch Lidartefakte bedingt

Im Reintonaudiogramm zeigte sich links eine pantonale mittel- bis hochgradige sensorineurale Schwerhörigkeit und rechts eine leichtgradige sensorineurale Hochtonschwerhörigkeit (Abb. 1a, b). Im Video-Kopfimpulstest fiel eine Unterfunktion des lateralen und posterioren Bogengangs links auf mit deutlich vermindertem Verstärkungsfaktor des vestibulookulären Reflexes mit Overt-Rückstellsakkaden (Gain des lateralen Bogengans links: 0,56; Abb. 1c, d). Aufgrund des akuten Schwindels war die Compliance des Patienten eingeschränkt.

## Bildgebende Diagnostik

**Abb. 2:**
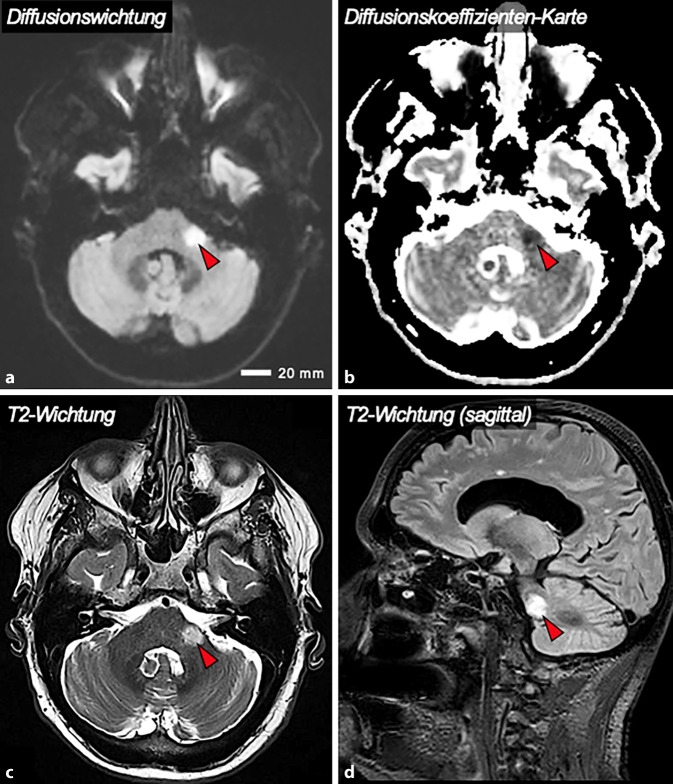
Bildgebende Diagnostik mittels Magnetresonanztomographie. Diese zeigte eine sphärische, diffusionsgestörte Läsion (*rote Pfeilspitze*) des linksseitigen Hirnstamms (**a–c**) und Kleinhirnstiels (**d**) unter Miteinbeziehung der Wurzeleintrittszone des Nervus vestibulocochlearis links. MR-Sequenzen: **a** Diffusionswichtung („Diffusion Weighted Imaging“, DWI); **b** Diffusionskoeffizienten-Karte („Apparent Diffusion Coefficient Map“, ADC-Map); **c** T2-Wichtung; **d** T2-Wichtung, „Fluid-Attenuation-Inversion-Recovery“(FLAIR)-Sequenz

Nach notfallmäßiger neurologischer Beurteilung wurde eine Magnetresonanztomographie (MRT) des Neurokraniums einschließlich einer MR-Angiographie durchgeführt. Diese zeigte einen zerebrovaskulären ischämischen Insult der kaudalen Medulla oblongata und des Kleinhirnstiels links unter Miteinbeziehung der Wurzeleintrittszone des Nervus vestibulocochlearis links (Abb. 2). Weiter bestand der Verdacht auf eine kurzstreckige Stenose des intrakraniellen Segments der *Arteria vertebralis* links. Eine dopplersonographische Kontrolle ergab eine hochgradig stenosierte *Arteria vertebralis* links bei ansonsten diskreter Plaquebildung der zuführenden Hirngefäße.

## Wie lautet Ihre Diagnose?

**Diagnose:** Zerebrovaskulärer Insult im Stromgebiet der Arteria cerebelli anterior inferior („Anterior Inferior Cerebellar Artery“, AICA) links

## Therapie und Verlauf

Der Patient wurde von der neurologischen Klinik umgehend zur stationären Überwachung und weiteren Diagnostik auf die Stroke Unit übernommen. Als Ätiologie des zerebrovaskulären Insults wurde eine am ehesten atherothrombotisch-ischämische Genese postuliert. Es wurden eine duale Thrombozytenaggregationshemmung mit Acetylsalicylsäure und Clopidogrel sowie zusätzlich eine plaquestabilisierende Therapie mit Atorvastatin initiiert. Nach unauffälliger Überwachung konnte der Patient mit deutlich gebesserten Schwindelbeschwerden ins häusliche Umfeld entlassen werden.

## Diskussion

Das akute vestibuläre Syndrom (AVS) ist definiert als klinisches Syndrom mit akut einsetzendem und andauerndem (d. h. nicht-episodischem) Schwindel, hinweisend auf eine neue, anhaltende Dysfunktion des vestibulären Systems, oft mit begleitender Übelkeit und Erbrechen, Nystagmus und posturaler Instabilität [1]. Falls kein ersichtlicher spezifischer Auslöser des AVS besteht, muss als wichtige Differenzialdiagnose ein zerebrovaskulärer Insult des vertebrobasilären (posterioren) Stromgebiets (Hirnstamm, Zerebellum und Innenohr) berücksichtigt werden [4]. Dabei weisen lediglich rund 50 % der Schlaganfallpatienten mit einem AVS fokal-neurologische Zeichen auf [5]. Auch kardiovaskuläre Risikofaktoren allein eignen sich nicht, um das Risiko einer zentralen Ursache zuverlässig abzuschätzen [1].

Bei Vorliegen eines AVS in Kombination mit einem Nystagmus hat sich das klinische HINTS-Untersuchungsprotokoll (Akronym für „*H*ead *I*mpulse Test“, „*N*ystagmus Type“, „*T*est of *S*kew“) seit 2009 als zuverlässige Methode zur Differenzierung eines peripheren von einem zentralen AVS etabliert [3–5]. Das Protokoll kann in den ersten 48 h nach Symptombeginn einen Schlaganfall zuverlässiger ausschließen als eine Magnetresonanztomographie [5]. „Red Flags“ für eine zentrale Genese sind in dieser Situation:

- ein normaler Kopfimpulstest (d. h. ohne Rückstellsakkade) und/oder
- ein zentrales Nystagmusmuster (horizontaler Blickrichtungsnystagmus, d. h. mit der Blickrichtung richtungswechselnder Nystagmus) und/oder
- eine vertikale Blickdeviation im wechselseitigen Abdecktest („Skew Deviation“).

Als weitere wichtige, unabhängige „Red Flag“ für eine zentrale Ursache eines AVS wurde dem HINTS-Untersuchungsprotokoll im Jahr 2013 ein akuter sensorineuraler Hörverlust hinzugefügt („HINTS plus“) [1–4], wodurch die Sensitivität des HINTS-Untersuchungsprotokolls nochmals gesteigert wird, da bei einer akuten kombinierten cochleären *und* vestibulären Funktionsstörung in bis zu 40 % der Fälle eine zentrale Ursache besteht [2]. Pathophysiologisch ist dies durch das Versorgungsgebiet der *Arteria cerebelli anterior inferior* zu erklären, welche über die *Arteria labyrinthi* die Cochlea und das Vestibularorgan versorgt. Falls bei einem zerebrovaskulären Insult im Stromgebiet der *Arteria cerebelli anterior inferior* nur das Innenohr, die Wurzeleintrittszone des Nervus vestibulocochlearis und/oder die Vestibularis-Kerngebiete betroffen sind, ist der vestibulookuläre Reflex gestört, was in einem pathologischen Kopfimpulstest resultiert [1, 2]. Weiter weist der Nystagmus oft periphere Charakteristika auf, und es findet sich keine vertikale Blickdeviation im Aufdecktest.

Somit weist das HINTS-Untersuchungsprotokoll wie im hier dargestellten Fall bei einem AVS mit cochleärer Beteiligung nur auf eine mögliche zentrale Ursache hin, wenn der akute Hörverlust als zusätzliches, *zentrales* Zeichen mitberücksichtigt wird (HINTS-plus-Untersuchungsprotokoll). Ein Hörverlust verbunden mit einem zentralen AVS ist naturgemäß immer sensorineural sowie in der Regel pantonal und mindestens mittelgradig ausgeprägt [2]. Zur Diagnose muss ein Reintonaudiogramm erfolgen, wobei in einer Notfallsituation mindestens Anamnese und die Stimmgabelversuche herangezogen werden sollen. Weitere einfach verfügbare diagnostische Mittel sind der Fingerreibeversuch oder Smartphone-Apps.

Weist das HINTS-plus-Untersuchungsprotokoll wie im hier dargestellten Fall eines AVS in (mindestens) einem Punkt auf eine zentrale Ursache hin, muss notfallmäßig eine neurologische Mitbeurteilung erfolgen [1]. Auch wenn oft keine akute Therapieindikation (z. B. zur systemischen Thrombolysetherapie) besteht, sollten die Patienten 24 bis 48 h stationär überwacht werden, da es in bis zu 25 % der Fälle zu einer neurologischen Verschlechterung kommen kann [3].

Die weitere Diagnostik (z. B. feingeschichtete MRT des posterioren Stromgebiets, Doppler-Untersuchung der hirnzuführenden Gefäße), Akuttherapie und ggf. Einleitung einer Sekundärprophylaxe (Thrombozytenaggregationshemmer, ggf. mit plaquestabilisierendem Statin) erfolgt unter Federführung der neurologischen Kollegen.

## Fazit für die Praxis

- Das HINTS-plus-Untersuchungsprotokoll in den ersten 48 h die zuverlässigste Methode, um eine zentrale Ursache eines akuten vestibulären Syndroms zu erkennen, wobei im Verdachtsfall eine Magnetresonanztomographie angeschlossen werden muss.
- Für den praktisch tätigen HNO-Arzt bedeutet dies: Findet sich bei einem akuten vestibulären Syndrom in Kombination mit einer gleichzeitig aufgetretenen sensorineuralen Hörminderung („Hörsturz mit vestibulärer Beteiligung“) keine offensichtliche otologische Ursache (z. B. eine Labyrinthitis), sollte differenzialdiagnostisch ein AICA-Infarkt erwogen werden – selbst bei pathologischem Kopfimpulstest.
- In diesen Fällen ist eine unmittelbare neurologische Vorstellung der Patienten indiziert.
- Das weitere Management der Patienten (z. B. stationäre Überwachung, Art und Zeitpunkt der Bildgebung, Akuttherapie, Sekundärprophylaxe) sollte unter neurologischer Federführung erfolgen.
